# Approaching Artificial Intelligence in Orthopaedics: Predictive Analytics and Machine Learning to Prognosticate Arthroscopic Rotator Cuff Surgical Outcomes

**DOI:** 10.3390/jcm12062369

**Published:** 2023-03-19

**Authors:** Anish G. Potty, Ajish S. R. Potty, Nicola Maffulli, Lucas A. Blumenschein, Deepak Ganta, R. Justin Mistovich, Mario Fuentes, Patrick J. Denard, Paul M. Sethi, Anup A. Shah, Ashim Gupta

**Affiliations:** 1 South Texas Orthopedic Research Institute (STORI Inc.), Laredo, TX 78045, USA; 2 The Institute of Musculoskeletal Excellence (TIME Orthopaedics), Laredo, TX 78041, USA; 3 School of Osteopathic Medicine, The University of the Incarnate Word, San Antonio, TX 78209, USA; 4 Department of Musculoskeletal Disorders, School of Medicine and Surgery, University of Salerno, 84084 Fisciano, Italy; 5 San Giovanni di Dio e Ruggi D’Aragona Hospital “Clinica Ortopedica” Department, Hospital of Salerno, 84124 Salerno, Italy; 6 Centre for Sports and Exercise Medicine, Barts and the London School of Medicine and Dentistry, Queen Mary University of London, London E1 4DG, UK; 7 School of Pharmacy and Bioengineering, Keele University School of Medicine, Stoke on Trent ST5 5BG, UK; 8 Department of Orthopaedics, School of Medicine, Case Western Reserve University, Cleveland, OH 44106, USA; 9 School of Engineering, Texas A&M International University, Laredo, TX 78041, USA; 10 Southern Oregon Orthopedics, Medford, OR 97504, USA; 11 Orthopaedic & Neurosurgery Specialists, Greenwich, CT 06905, USA; 12 Kelsey-Seybold Clinic, Houston, TX 77584, USA; 13 Future Biologics, Lawrenceville, GA 30043, USA; 14 BioIntegrate, Lawrenceville, GA 30043, USA; 15 Regenerative Orthopaedics, Noida 201301, Uttar Pradesh, India

**Keywords:** machine learning, artificial intelligence, orthopaedics, arthroscopic rotator cuff repair, functional outcomes, predictive modelling, American Shoulder and Elbow Surgeons (ASES) score

## Abstract

Machine learning (ML) has not yet been used to identify factors predictive for post-operative functional outcomes following arthroscopic rotator cuff repair (ARCR). We propose a novel algorithm to predict ARCR outcomes using machine learning. This is a retrospective cohort study from a prospectively collected database. Data were collected from the Surgical Outcome System Global Registry (Arthrex, Naples, FL, USA). Pre-operative and 3-month, 6-month, and 12-month post-operative American Shoulder and Elbow Surgeons (ASES) scores were collected and used to develop a ML model. Pre-operative factors including demography, comorbidities, cuff tear, tissue quality, and fixation implants were fed to the ML model. The algorithm then produced an expected post-operative ASES score for each patient. The ML-produced scores were compared to actual scores using standard test-train machine learning principles. Overall, 631 patients who underwent shoulder arthroscopy from January 2011 to March 2020 met inclusion criteria for final analysis. A substantial number of the test dataset predictions using the XGBoost algorithm were within the minimal clinically important difference (MCID) and substantial clinical benefit (SCB) thresholds: 67% of the 12-month post-operative predictions were within MCID, while 84% were within SCB. Pre-operative ASES score, pre-operative pain score, body mass index (BMI), age, and tendon quality were the most important features in predicting patient recovery as identified using Shapley additive explanations (SHAP). In conclusion, the proposed novel machine learning algorithm can use pre-operative factors to predict post-operative ASES scores accurately. This can further supplement pre-operative counselling, planning, and resource allocation. Level of Evidence: III.

## 1. Introduction

The optimal management of shoulder disorders depends on recognizing the natural history of disability caused by an injury and the anticipated outcomes after operative treatment when this is indicated. Clinical and patient-reported outcome measures are critical to truly understand post-operative function and monitor the progress of an effective care plan [1]. Using machine learning (ML), the goal of this study was to analyse such factors to determine the factors most predictive for successful outcomes. ML is a novel field of study that employs computer algorithms and statistical analysis to determine complex trends and patterns in the data that may not be easily discernible by humans. ML uses data to build empirical/statistical models to describe the behaviour of a system [2].

As such, there is a growing body of literature on machine learning to analyse data and answer clinical questions for both the diagnosis and prognostication of rotator cuff tears [3,4,5]. Recent reviews have demonstrated the wide range of potential applications, from the analysis of ultrasound to diagnose rotator cuff tears to the characterization of rotator cuff fatty degeneration on CT (computer tomography) scans [3,5]. Conversely, other studies have attempted to develop a clinical prediction tool to forecast the chance of complications versus clinical improvement following repair [4]. However, while exciting, there is much room for improvement regarding the application and accuracy of such ML models [5].

Focusing on patients with operative rotator cuff pathology, we developed a novel algorithm to predict arthroscopic rotator cuff repair (ARCR) outcomes. We examined pre-operative and post-operative American Shoulder and Elbow Surgeons (ASES) score. The ASES score is a widely reported and validated patient-reported outcome measure (PROM) applicable to all patients with shoulder pathologies and independent of their specific diagnosis [6,7].^⁠^ Through machine learning, we sought to understand whether specific pre-operative characteristics could ultimately predict recovery.

Rotator cuff tears are an ideal musculoskeletal condition to study with machine learning. Many factors influence treatment and outcomes, complicating the ability of surgeons to predict outcomes reliably. Secondly, the burden of disease is substantial: each year, an estimated 250,000 people in the U.S. suffer rotator cuff tears, resulting in 3–4 billion dollars spent annually [8].

As a result, surgeons may be economically “punished” for taking on more complex cases with the potential for poorer outcomes, and such policies may act as a barrier to care for those who need it most.

However, these questions cannot be adequately answered with conventional statistical methods such as simple linear regression, as large volumes of data can be scattered. Machine learning (ML) algorithms can overcome these limitations, improve prediction accuracy, and reduce the margin of error between actual and predicted data [2,9,10,11,12]. This approach was recently applied in orthopaedics to optimize the number of questions in the Knee Injury and Osteoarthritis Outcome Score (KOOS) activities of the daily living questionnaire following knee surgery [2].

The goal of this study is to use ML in prospectively collected pre- and post-operative data of patients who underwent ARCR to develop a novel algorithm to predict arthroscopic rotator cuff outcomes. We hypothesized that ML algorithms could be used to predict 3-, 6-, and 12-month post-operative ASES scores for patients who underwent rotator cuff repair.

## 2. Materials and Methods

### 2.1. Study Design

We performed a retrospective review of prospectively collected data of patients who underwent ARCR performed by several surgeons between April 2011 and April 2019. The surgical technique was based on the surgeon’s preference. As the data were collected for a multi centric database, all surgeries were performed with single- or double-row suture bridge construct with a configuration of 3 medial 2 lateral, 2 medial 2 lateral, 1 medial 2 lateral, and 2 medial 1 lateral for the double-row fixation and 3 and 2 anchors for the single row construct. Data for analysis was extracted from the Surgical Outcome System (SOS) global registry, an international patient-reported outcome database maintained by Arthrex. No institutional review board (IRB) approval was required, as SOS global registry is IRB approved and adheres to Health Insurance Portability and Accountability Act (HIPAA) regulations. All SOS global registry users have access to the shared deidentified data.

We included patients who had fully documented demographic and surgical data. Patients who did not complete pre-operative, 3-, 6-, and 12-month post-operative ASES surveys were excluded. Additional exclusion criteria included patients who underwent revision surgery, those who lacked complete follow-up at the specified time points, and patients with incomplete questionnaires. The dependent (or target) variables for this study were 3-, 6-, and 12-month post-operative ASES scores. Several patient- and surgery-related independent variables were considered for this multivariate analysis to better understand their impact on the target variables. Patient-related factors examined were gender, age, BMI, tobacco use, and past medical history of diabetes. Surgical-related factors included the number of tendons torn, tendon quality, Cofield tear size, retraction stage, tear shape, medial anchor type, number of medial knotless anchors, number of medial suture anchors, lateral anchor type, number of lateral knotless anchors, number of lateral suture anchors, pre-operative visual analogue pain score (VAPS), pre-operative ASES score, and the year of operation.

The binary features in the dataset (gender, tobacco use, and history of diabetes) were encoded as “0” and “1”. The other categorical features in the dataset were converted to numerical values based on domain understanding to improve model predictions. The following encoding was used: tendon quality (poor: 1, fair: 2, good: 3, excellent: 4); Cofield tear size (small (<1 cm): 1, medium (1–3 cm): 2, large (3–5 cm): 3, massive (> 5 cm): 4); retraction stage (stage I: 1, stage II: 2, stage III: 3, stage IV: 4); tear shape (L-shaped posterior: 1, L-shaped anterior: 2, U-shaped: 3, avulsion/crescent: 4, massive contracted: 5, longitudinal: 6); anchor type (suture anchor: 1, knotless anchor: 0, tenodesis screw: 0).

### 2.2. Data Preparation and Model Building

Data processing, analysis, and ML model building were performed using Python 3.7.4 (http://www.python.org accessed on 1 December 2022). Python packages such as matplotlib, NumPy, Pandas, and Scikit-learn were used for data wrangling, statistical analysis, visualization, and ML model building [13]. The surgical outcomes model is a multi-target regression problem, as the goal is to predict patient recovery at multiple time points (3, 6, and 12 months) after surgery using pre-operative information. To achieve this, the multioutputregressor function from the scikit-learn library in Python was used to fit multiple target variables. Cross-validation (CV) is a de facto standard to estimate model prediction errors and the most popular approach for model selection and hyperparameter tuning [14]. K-fold cross-validation involves partitioning the dataset into k equal-sized subsets (or folds), training the ML model on all but one subsets (i.e., k-1 subsets), and then evaluating the model on the held-out subset. This process is then repeated k times with a different subset held out each time.

The data were randomly split into two sets: a training set with 80% data and a test set with the remaining 20% data. The 80/20 data split is a commonly used ML method and was chosen accordingly [15]. The ML model was trained using the training data, and the model’s performance was confirmed on the test data. Each feature, prior to model building, was scaled (forcing the mean to 0 and scaling the variance to 1) to help to better interpret the model results. Several machine learning models (linear regression, ridge regression, lasso, support vector regression, k-nearest neighbour, random forest, and XGBoost) were evaluated in this study, and the best model was selected based on 10-fold CV error (Table 1). Although linear regression, ridge regression, and lasso had lower RMSE, XGBoost was chosen as the “best” model for further refinement based on acceptable root-mean-square error (RMSE) and normally distributed errors (less bias in model predictions). Moreover, linear regression, ridge regression, and lasso models highly weighted “gender” as the most important predictor of post-operative ASES, which medically seems highly unlikely. Therefore, XGBoost was chosen as the “best” model for this work. Hyperparameter tuning for the XGBoost model was performed to identify the “best” model based on minimizing the RMSE through 5-fold cross-validation [16]. This was accomplished by searching over a grid space of select key XGBoost hyperparameters (learning_rate: 0.001, 0.005, 0.01, 0.1; max_depth: 6, 8, 10; n_estimators: 200, 400, 500, 600, 700; min_child_weight: 0.5, 1, 2; colsample_bytree: 0.3, 0.5) using the “GridSearchCV” object in Scikit-learn. The set of XGBoost hyperparameters that resulted in the lowest cross-validation error were as follows: n_estimators = 400, learning_rate = 0.01, max_depth = 6, min_child_weight: 2, and colsample_bytree = 0.5. The performance of the “best” model was then evaluated on the test dataset to gauge its performance on this blind, held-out data.

**Figure 1:**
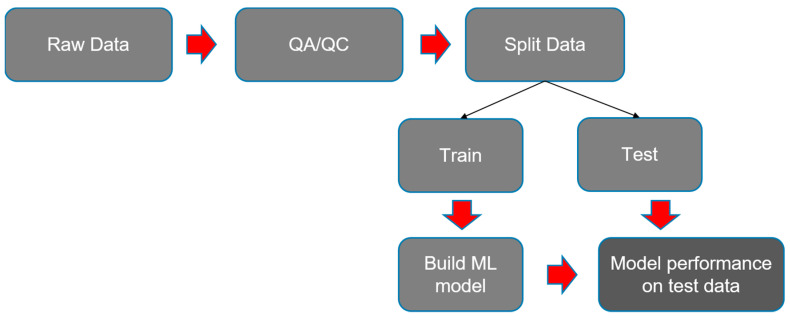
Simple illustration of various steps and their dependence on the machine learning process description.

To better understand model predictions, the SHapley Additive exPlanations (SHAP) method was used to explain global feature importance and individual predictions [17]. SHAP values were first obtained using the TreeExplainer method to explain every prediction of the XGBoost model. The next step involved plotting the explanations using the “summary_plot” method. Explanation of individual predictions was performed using the “force_plot” method within the SHAP library. To summarize, the methods involved are shown in Figure 1 through a simple illustration.

### 2.3. Data Analysis

**Figure 2:**
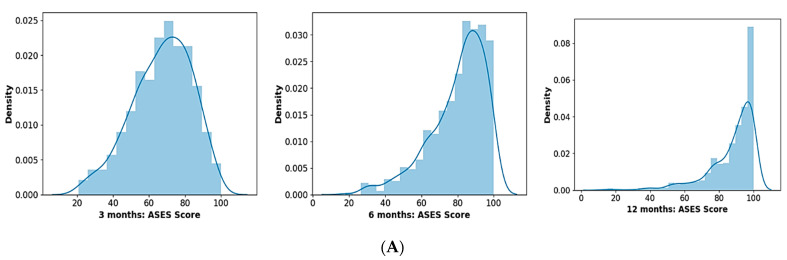

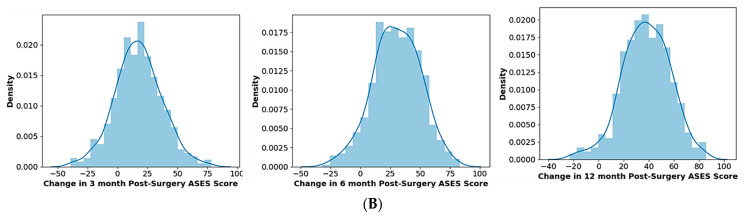
Histogram of (**A**) 3-, 6-, and 12-month post-operative ASES scores and (**B**) change in 3-, 6-, and 12-month post-operative ASES scores compared to pre-operative ASES score.

Distributions of raw data were analysed for symmetry and skewness. The data were non-symmetric and left-skewed with a skewness of −0.45, −1.12, and −1.92 for the 3-, 6-, and 12-month post-operative ASES scores, respectively, meaning that the tail on the left side (or lower ASES scores) of the distribution is considerably drawn out compared to the right tail (or higher ASES scores) of the distribution (Figure 2). This is not surprising since the expectation is that most patients would experience an improvement in ASES scores after surgery. For reference, a symmetric distribution such as the normal distribution (bell curve) has a skewness of 0. Since the target (or independent) variables (i.e., post-operative ASES scores) are left-skewed, the machine learning model is trained on an imbalanced dataset and is less likely to accurately predict the outcome in patients with low post-operative ASES scores. Similarly, if the target variable were right-skewed, the model would be less likely to correctly predict cases with high post-operative scores. To reduce target variable skewness and improve model predictions, a mathematical transformation was performed by subtracting the pre-operative score from the post-operative score. This transformed variable demonstrates the improvement in post-operative ASES scores compared to the pre-operative scores. The post-transformation distributions for 3, 6, and 12 months post operation had a corresponding skewness of 0.06, −0.14, and −0.24. In other words, the skewness is closer to zero, indicating that these distributions are more symmetric and closer to a normal distribution. Other mathematical transformations such as log, square, and square root were not as effective on this dataset.

The model building and model selection steps were undertaken using the transformed target variable described in the Methods section (Section 2.2 data preparation and model building). Root-mean-square error (RMSE) is a measure of the standard deviation of the prediction errors and is a commonly used heuristic to evaluate machine learning models. The average RMSE, calculated using 10-fold CV, for the various ML models (with default hyperparameters) studied in this work was between 15.3–17.2 for the 3-month post-operative ASES score predictions. For the XGBoost model, the RMSE along with 95% confidence interval calculated using 10-fold cross validation of the training data was 15.90 (95% CI: 14.80–17.00), 16.36 (95% CI: 15.70–17.02), and 14.60 (95% CI: 12.84–16.36) for 3, 6, and 12 months post operation, respectively [18]. The RMSE for the test dataset prediction was 16.50, 14.75, and 12.94 for 3, 6, and 12 months post operation, respectively (Table 2).

**Figure 3:**
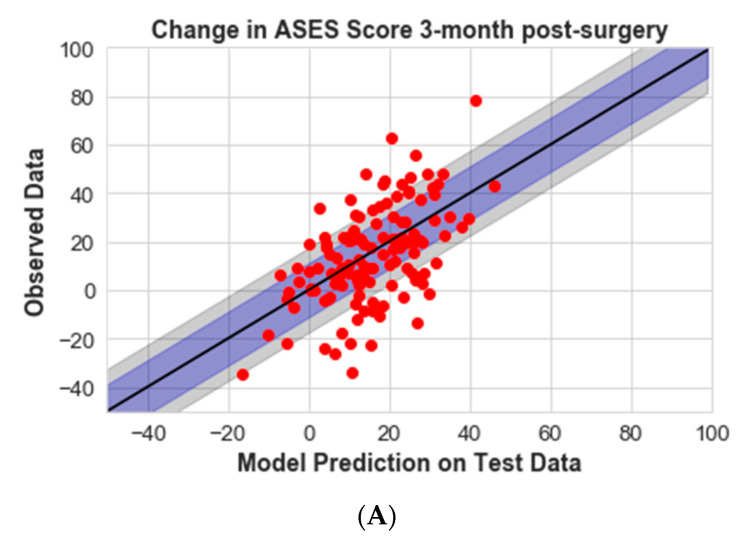

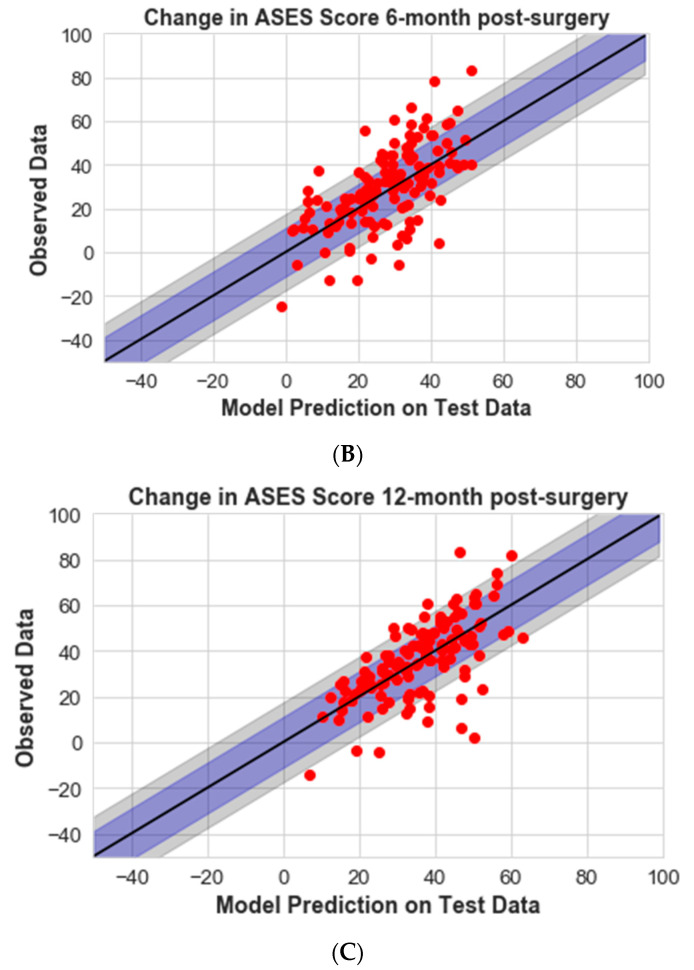
Parity plot of observed and predicted change in ASES score for 3- (**A**), 6- (**B**), and 12-month (**C**) post-operative follow-up relative to the pre-operative ASES score for the test dataset. The shaded blue region is the MCID, and the shaded grey region is the SCB.

The minimal clinically important difference (MCID) and substantial clinical benefit (SCB) values were obtained from the literature and are also shown in Figure 3 [19]. MCID and SCB were used to characterize the extent of error in model predictions. MCID, in this case, is the smallest change in ASES score that a patient would perceive as meaningful. While MCID is defined as the minimum improvement threshold, SCB indicates a substantial change in clinical state as perceived by the patient.

SHAP is a widely used, game-theory-based approach to explain global and local model behaviours [16]. SHAP was used to indicate which features are the most predictive of outcomes. SHAP mean value was used to rank variables or features by highest to least impact on the target variable (i.e., change in ASES score) for the dataset.

## 3. Results

A total of 4729 patients who underwent ARCR were identified. There were 631 patients between the ages of 24–83 years who met inclusion criteria for analysis (mean age 61.5 years). Of the 631 patients, 362 were males (57%), and 269 were females (43%).

**Figure 4:**
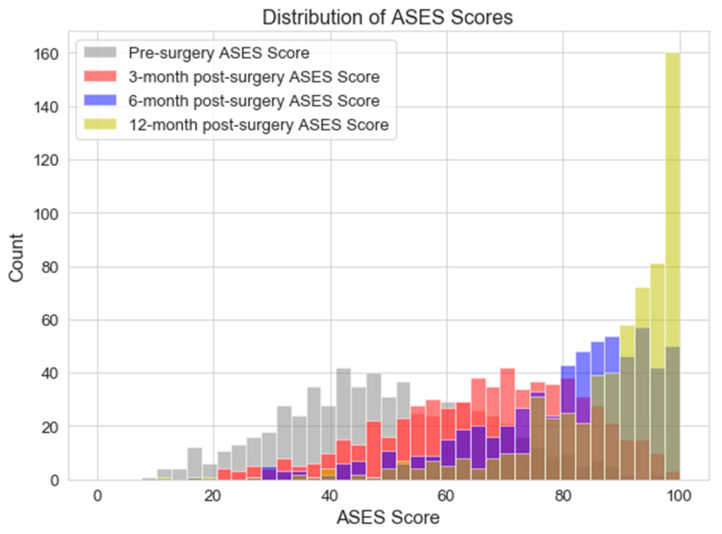
Progressive distribution of the pre-operative, 3-, 6-, and 12-month post-operative ASES scores.

Pre-operative ASES score distribution had a mean and standard deviation of 50 and 17, respectively (Figure 4). The mean post-operative scores increased with recovery time, with the data demonstrating a mean and standard deviation for the 12-month post-operative ASES scores of 87 and 12, respectively, demonstrating a mean improvement in functional outcomes after surgery.

The model demonstrated reasonable performance in predicting recovery progression that healthcare providers could use in their decision-making process. A substantial number of the test dataset predictions using the XGBoost algorithm were within the MCID and SCB thresholds: 69% of the 12-month post-operation predictions were within MCID, while 87% were within SCB, which correlates with the clinical findings of ASES score improvement (Figure 3). The percentage of patients (in the test dataset) predicted within the MCID for 3, 6, and 12 months post operation was 52%, 54%, and 69%, respectively. Similarly, the percentage of patients predicted within SCB for 3, 6, and 12 months post operation was 73%, 78%, and 87%, respectively. The scatter plots comparing model prediction and observed data for 3, 6, and 12 months post operation are demonstrated in Figure 3.

**Figure 5:**
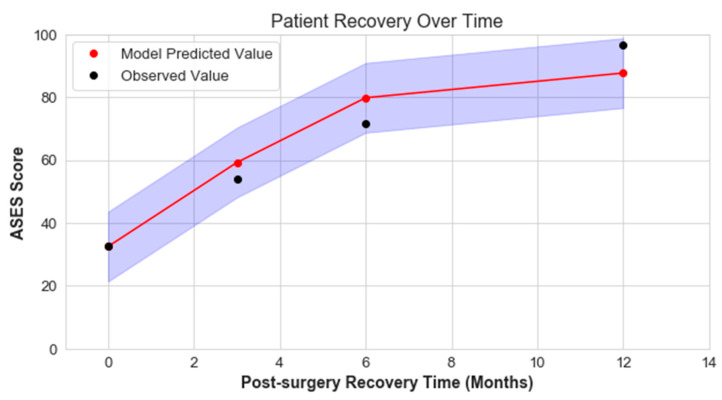
Model predicted 3-, 6-, and 12-month post-operative ASES scores (red) based on pre-operative information for a patient from the test dataset. The observed ASES score (black) is also shown. The shaded region (blue) indicates the bounded area for the MCID value of 11.1.

Based on pre-operative information, the model was able to predict 3-, 6-, and 12-month post-operative ASES scores following ARCR (Figure 5). Although the predictions did not exactly match the actuals, the predicted improvement in ASES score lies within the MCID value.

Unlike linear regression models that provide statistical significance (*p*-value) of each feature as an output, black box ML models (e.g., random forest, XGBoost) output a feature importance score that ranks the relative contribution of each feature towards predicting the target variable. For feature importance, the black box models not only account for main effects but also account for interaction effects between different features. It is important to understand the trade-off between model accuracy and model interpretability. Simple models (e.g., linear regression) have good interpretability but may not have good accuracy. By contrast, complex models usually have better accuracy but poor interpretability (i.e., lack clear explanation of why the model made such a prediction). ML practitioners, having realized the importance of solving this problem, are actively working on various methods to address model interpretability [20]. SHAP is one such approach.

**Figure 6:**
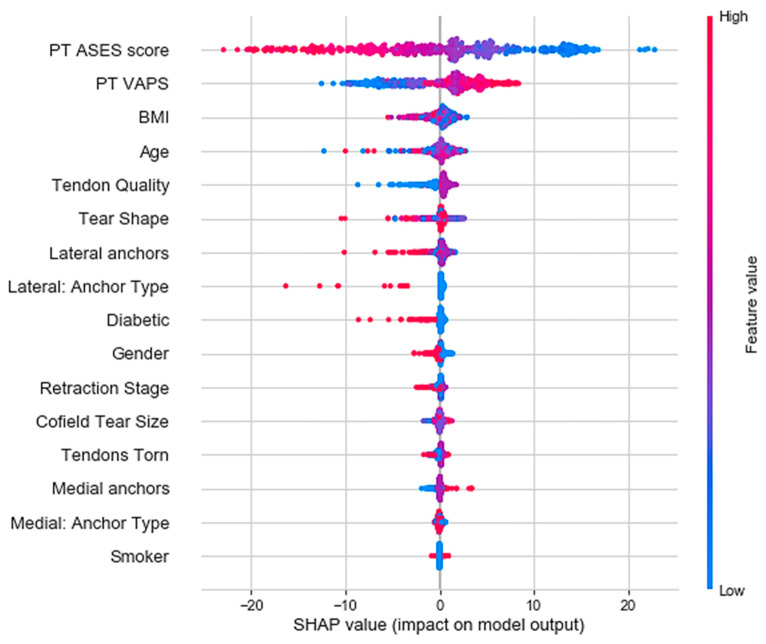
Relative importance of various features on the prediction of 12-month post-operative ASES score.

As shown in the SHAP summary values (Figure 6), the features most predictive of post-operative ASES score included the pre-operative ASES score, pre-treatment VAPS, BMI, patient age, and tendon quality. The least predictive factor was patient smoking status (Figure 6). The SHAP summary plot also shows both the positive and negative relationships of the predictor variables. High pre-operative ASES scores negatively impacted SHAP values. In other words, a patient starting with a high pre-operative ASES score did not have as high of a potential to increase their score further and therefore demonstrated a smaller magnitude of change in the ASES score. In contrast, a patient starting with a low pre-operative ASES score has a larger potential for improvement. Similar conclusions can be drawn for the pre-operative VAPS. Additionally, poor tendon quality leads to slower recovery.

### Case Example

**Figure 7:**
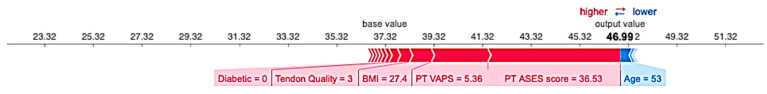
Contribution of various features obtained using SHAP on the prediction of change in 12-month post-operative ASES score for a test dataset patient.

Note that these SHAP summary results are global, i.e., overall trends observed based on this dataset. Interestingly, SHAP can also be used to understand the impact of each factor at an individual patient level. Figure 7 shows the explanation plot generated using the SHAP “force plot” method for one patient in the test dataset. The plot indicates the individual contribution of various factors that lead to the model prediction: improvement in ASES score of 46.99. The base score of 37.32 (the average change in 12-month post-operative ASES score) is also shown in the plot. As shown in Figure 7, the largest contributions in increasing the score come from the pre-operative ASES score and pre-operative VAPS. In addition, the plot attributes having good tendon quality and lower (than average in this dataset) BMI suggest better recovery.

## 4. Discussion

One of the key findings from this study was that ML could forecast post-operative patient recovery over time using pre-operative factors (Figure 5). We identified pre-operative ASES score, pre-operative VAPS, BMI, age, and tendon quality as key factors impacting patient outcomes (Figure 6). These findings provide a better understanding of the factors influencing surgical outcomes, leading to better informed consent and personalized patient care with data-driven expectations for post-operative recovery.

The application of ML in orthopaedics has recently increased [2,9,10,11,12,21]. While the number of orthopaedic studies utilizing ML is still limited, studies from other fields demonstrate its capability to surpass human performance [22,23,24]. Several orthopaedic studies demonstrate ML can optimize the use of pre-operative assessments and accurately predict the likelihood of patients achieving MCID or SCB in PROMs postoperatively [2,10,21].

One might expect that pre-operative assessment scores would be inversely related to post-operative outcomes; that is, that higher pain and lower functional scores pre-operatively would predict worse post-operative outcomes. Likewise, several studies demonstrate this to be the case [25,26,27,28]. Conversely, we found lower pre-operative ASES and VAP scores to be the most significant predictors of post-operative ASES score improvement, with higher pre-operative values negatively influencing post-operative scores. This finding is consistent with Jenssen et al. [29], who analysed factors predictive of post-operative functional outcomes following arthroscopic rotator cuff surgery. They found pre-operative pain scores to be negatively associated with post-operative shoulder function following shoulder arthroplasty. This could be attributed to the fact that patients with worse pre-operative assessments have the greatest potential for improvement. It could also be that patients with worse pre-operative pain can better appreciate their improvements, which reflects the more improved post-operative assessments.

In the present study, individuals with a lower (than average in this dataset) BMI demonstrated better recovery. However, no significant association was found between diabetes mellitus and outcomes. The effects of high BMI, diabetes mellitus, and dyslipidaemia have been previously studied with varied findings. Several studies found that these variables predict worse clinical outcomes, recovery, and tendon healing following rotator cuff injury [30,31,32,33]. Conversely, other studies demonstrated no association of BMI with post-operative outcome scores following ARCR [29,34].

Younger age at the time of repair was positively associated with improved outcomes, though the prior literature on this topic is conflicting. Generally, younger age correlates with more successful recovery following rotator cuff repair [35,36]. Likewise, older age is negatively associated with successful tendon healing, longer recovery time, and increased risk of re-tear following ARCR [37,38,39]. Other studies demonstrate that when accounting for fatty infiltration, bone mineral density, or retraction of the rotator cuff tendon, there is no independent association between age and rotator cuff healing [40,41,42]. Furthermore, in other studies, younger age is associated with worse pain and functional outcomes [27,29,43].

In the present investigation, lower pre-operative tendon quality correlated with lower post-operative ASES scores. However, tendon tear size, the number of tendons torn, tear shape, and retraction stage was not strongly associated with a poor outcome. The literature also demonstrates that several measurements of pre-operative tendon quality are associated with worse post-operative outcomes [36]. A larger pre-operative rotator cuff tear size negatively impacts healing, recovery time, functional outcomes, and rate of retear [8,32,38,44,45,46]. Additionally, tendon retraction and fatty infiltration demonstrate a negative impact on healing [34]. Likewise, studies demonstrate that patients with multiple tendon injuries are more likely to develop a rotator cuff defect [47].

Our results did not demonstrate a strong association between gender and post-operative ASES scores, another area of conflicting findings in the literature. Several studies show female sex to be associated with worse post-operative quality of life, mental health, pain, and functional assessments [26,27,48,49,50,51]. Conversely, other studies demonstrate that gender does not influence post-operative outcomes [39].

We also did not identify a strong association between tobacco use and post-operative ASES scores. This finding correlates to a prior study demonstrating that tobacco use is not associated with post-operative structural failure following rotator cuff repair [52]. Nonetheless, other studies found that smoking is associated with an increased risk of rotator cuff tears and tear size and worse post-operative clinical outcomes [29,53]. Tobacco use is a modifiable risk factor, and until more definitive data are produced, it is feasible to recommend cessation for the purposes of undergoing ARCR.

## 5. Limitations

Our results are encouraging, but we are aware of the limitations of the present study. While our sample size is relatively large, there still could be differences between predicted and actual results when applying the model to a real-world population. The model performance (i.e., RMSE) could be further improved by including additional input variables (e.g., compliance with physical therapy protocols and recovery exercises, pain management, and psychological factors) that could impact patient recovery. Additionally, the predicted values from the model may also not represent various regions around the world with different demographics, gender, or ethnic groups. Other features such as patient behavioural factors, medical risk factors, and chronic medical conditions before surgery should also be considered to more accurately predict patient recovery. Additionally, there could also be a recall bias when patients are reporting their outcome questionnaire. However, with more input data, new variables, and model-building refinement, this approach can significantly help surgeons customize their care plans.

One potential application of the model is for surgeons to share the predicted recovery profile with their patients before surgery, thereby setting baseline expectations (Figure 7). Furthermore, the surgeon may run different scenarios—manually or algorithmically—to identify the best course of action for each patient along with any modifiable risk factors. This will allow surgeons to better modulate treatment and rehabilitation techniques with greater confidence.

## 6. Conclusions

This proposed novel ML algorithm can predict the post-operative ASES scores after ARCR with satisfactory accuracy. While not intended for use in isolation, this model can be used as a critical tool for physicians to formulate better decisions and provide customized, evidence-based care for every patient. In addition, the model may be able to identify high-risk patients early on and enables surgeons and caregivers to give additional focus to such patients.

## Figures and Tables

**Table 1:** Root-mean-square error (RMSE) and 95% confidence interval (CI) calculated using 10-fold cross-validation to help identify “best” machine learning model.

Algorithm	10-Fold CV RMSE for 3-Month Post-Operative ASES	95% CI
LASSO	15.25	14.36–16.14
Ridge Regression	15.31	14.47–16.16
Linear Regression	15.32	14.47–16.16
XGBoost	15.90	14.80–17.00
Random Forest	16.35	15.40–17.31
K-Nearest Neighbour (KNN)	17.02	16.04–18.01
Support Vector Regression	17.20	16.00–18.40

**Table 2:** Root-mean-square error (RMSE) calculated using 10-fold cross-validation for train and test datasets at different post-operative timelines.

Post-Operative Time	Train RMSE (10-Fold CV) in ASES	Test RMSE in ASES
3 months	15.90	16.50
6 months	16.36	14.75
12 months	14.60	12.94

## Data Availability

All data are contained within the manuscript.
